# Self-Assembly of Hydrofluorinated Janus Graphene Monolayer: A Versatile Route for Designing Novel Janus Nanoscrolls

**DOI:** 10.1038/srep26914

**Published:** 2016-05-31

**Authors:** Yakang Jin, Qingzhong Xue, Lei Zhu, Xiaofang Li, Xinglong Pan, Jianqiang Zhang, Wei Xing, Tiantian Wu, Zilong Liu

**Affiliations:** 1 State Key Laboratory of Heavy Oil Processing, China University of Petroleum, Qingdao 266580, Shandong, P. R. China; 2 College of Science, China University of Petroleum, Qingdao 266580, Shandong, P. R. China; 3 Nano-Science Center and Department of Chemistry, University of Copenhagen, Copenhagen, DK-2100, Denmark

## Abstract

With remarkably interesting surface activities, two-dimensional Janus materials arouse intensive interests recently in many fields. We demonstrate by molecular dynamic simulations that hydrofluorinated Janus graphene (J-GN) can self-assemble into Janus nanoscroll (J-NS) at room temperature. The van der Waals (vdW) interaction and the coupling of C-H/π/C-F interaction and π/π interaction are proven to offer the continuous driving force of self-assembly of J-GN. The results show that J-GN can self-assemble into various J-NSs structures, including arcs, multi-wall J-NS and arm-chair-like J-NS by manipulating its original geometry (size and aspect ratio). Moreover, we also investigated self-assembly of hydrofluorinated J-GN and Fe nanowires (NWs), suggesting that Fe NW is a good alternative to activate J-GN to form J-NS. Differently, the strong vdW interaction between J-GN and Fe NW provides the main driving force of the self-assembly. Finally, we studied the hydrogen sorption over the formed J-NS with a considerable interlayer spacing, which reaches the US DOE target, indicating that J-NS is a promising candidate for hydrogen storage by controlling the temperature of system. Our theoretical results firstly provide a versatile route for designing novel J-NS from 2D Janus nanomaterials, which has a great potential application in the realm of hydrogen storage/separation.

With remarkably interesting surface activities, Janus materials, consisting of two different compositions half by half in one unit, have attracted much attention in recent decades^1^,^2^,^3^,^4^,^5^, which hold diverse potential applications as novel sensors, self-propellant motors, solid surfactants and building blocks for superstructures. Till now, various Janus materials with diverse shapes have been synthesized, e.g. zero-dimensional (0D) spheres^1^, one-dimensional (1D) rods^2^, two-dimensional (2D) disks/sheets^4^, wherein, 2D Janus materials arouse intensive interests in the field of emulsification for their large adsorption energy and highly confined rotation at the oil-water interface^5^,^6^. Graphene (GN), a monoatomic layer of *sp*^2^-hybridized carbon atoms with honeycomb structure, is always considered as the most promising 2D materials since discovered from a graphite crystal by a simple mechanical cleavage in 2004^7^, due to its remarkable electronic, mechanical and thermal properties^8^,^9^,^10^. Janus graphene (J-GN), one of a great variety of graphene derivatives, has been predicted theoretically and prepared experimentally, which achieves asymmetrically covalent functionalization with different functional groups on its two sides^11^,^12^,^13^,^14^. It has been proven clearly that the covalent functionalization is an effective path for tuning the band gap of GN, such as hydrogenation and fluorination, which avoids pristine GN’s lack of band gap and extends its further application in macro-electronics^9^. Via covalent chemistry, the *sp*^2^-hybridized carbon atoms are transformed into *sp*^3^-hybridized ones because of the formation of a single bond with external doping groups, which contributes to opening a wider band gap of GN^4^.

Experimental and theoretical studies have exhibited various J-GNs^11^,^12^,^13^,^14^,^15^,^16^,^17^,^18^, such as H/F type J-GN, Cl/phenyl type J-GN, etc. From experimental aspects, Zhang *et al.* have experimentally achieved J-GN using a two-step surface covalent functionalization, co-grafting asymmetrically of halogen and aryl/oxygen-functional groups on each side^11^. Moreover, the asymmetric functionalization of the monolayer graphene by semiconducting and metallic nanostructures at the polarizable liquid/liquid interface was also successfully achieved using low-cost and solution chemistry-based two-step functionalization^14^. Recently, the asymmetric structure of Janus graphene oxide (GO) was also achieved by the graft of amino-containing chemical groups on the outer surface, which could effectively stabilize an oil-in-water Pickering emulsion due to its amphiphilic structure^15^. From theoretical aspects, Yang *et al.* have theoretically demonstrated a novel Janus-type graphene with a robust non-zero gap by asymmetrical modification with covalently bonded H, F, Cl, and Br on opposing sides of GN sheet^11^. Singh *et al.* suggested theoretically the existence of a stoichiometric derivative of graphene, hydro-fluorinated graphene, with fluorine and hydrogen atoms alternately attached to carbon atoms in a chair-like configuration^16^. Moreover, Li *et al.* have investigated that the individual hydro-fluorinated GN monolayer is semiconducting with a direct energy gap of 2.82 eV^12^. In a word, the J-GN draws great attentions because it provides an ideal 2D bifacial Janus nanostructure to study asymmetric chemistry and further applications. However, due to the existence of substrates during the synthesis, the properties of freestanding J-GN monolayer are rarely investigated. Therefore, it is well worth to investigate the properties of freestanding J-GN and explore its potential applications in various fields.

Based on the novel hydrofluorinated J-GN, we demonstrate that hydrofluorinated J-GN monolayer can self-assemble into Janus nanoscroll (J-NS) structures at room temperature, which is mainly controlled by its geometry (size and aspect ratio). In the previous reports, the formation of carbon nanoscroll (CNS) has been demonstrated by rolling up a single GN sheet^19^. Due to the excellent mechanical and electronic properties, there are many applications of CNS in the field of gas storage/separation^20^, water purification^21^, nanoactuators^19^,^22^, etc. However, the study of the functionalizations of CNS is seldom reported. According to the reported preparation methods of CNS, such as high-energy ball milling^23^, chemical routes^24^,^25^, and defect-induced method^26^, it is still difficult to synthesize high-quality CNS and implement its functionalization. Herein, the formation of J-NS realizes the two-side doping of CNS and makes the further application of doped CNS conceivable in the realm of gas adsorption/separation, nano-reaction, etc.

In the present study, our interests in the self-assembly of J-GN is threefold. Firstly, due to the existence of substrates in the process of synthesis, the properties of freestanding J-GN monolayer are rarely investigated. Herein, freestanding hydrofluorinated J-GN monolayer, one of the magic 2-D Janus materials, is studied comprehensively. The theoretical results obtained in this work would shed important light on its properties of self-scrolling. Secondly, a versatile route for fabricating high-quality J-NS heterostructure and other novel nanostructures is proposed. Various types of J-GN have been synthesized using step-by-step functionalization, which makes diverse freestanding J-GNs conceivable. Therefore, we can obtain diverse J-NSs from the freestanding J-GNs using the proposed method. Compared with the conventional CNS^27^, the J-NS heterostructure proposed here are brand-new, which are hydrofluorinated on two sides of carbon and may exhibit potential applications in gas separation/storage. Thirdly, it is very interesting that J-NS provides considerable space for gas storage, especially for H_2_ sorption, and separation. In addition, this work provides a new avenue to designing J-NS from 2D Janus materials and extends further application of 2D J-GN.

## Results

### J-GN supercell model

**Figure 1:**
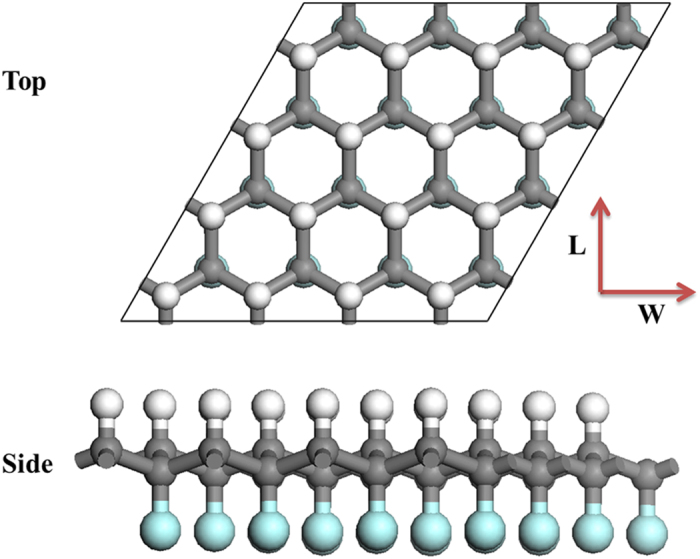
Geometric structures of J-GN supercell with zigzag structures along the width edges (W) and armchair structures along the length edges (L). White, gray, and green balls represent hydrogen, carbon and fluorine atoms, respectively.

A hydrofluorinated J-GN supercell is shown in Fig. 1, which is the most stable structure in the H and F co-grafting GN^12^,^13^. Clearly, the unique J-GN possesses the discontinuous co-grafting of H and F atoms in the each side of GN. Due to the chemisorption of H and F atoms, all carbon atoms are tuned from the *sp*^2^ to *sp*^3^ hybridization, and the perfect 2D plane displays some wrinkles from side view. In each simulation, J-GN has zigzag structures along the width edges (W) while armchair structures along their length edges (L). The aspect ratio of J-GN sheet is defined as r = L/W.

### Self-assembly of Hydrofluorinated J-GN

**Figure 2:**
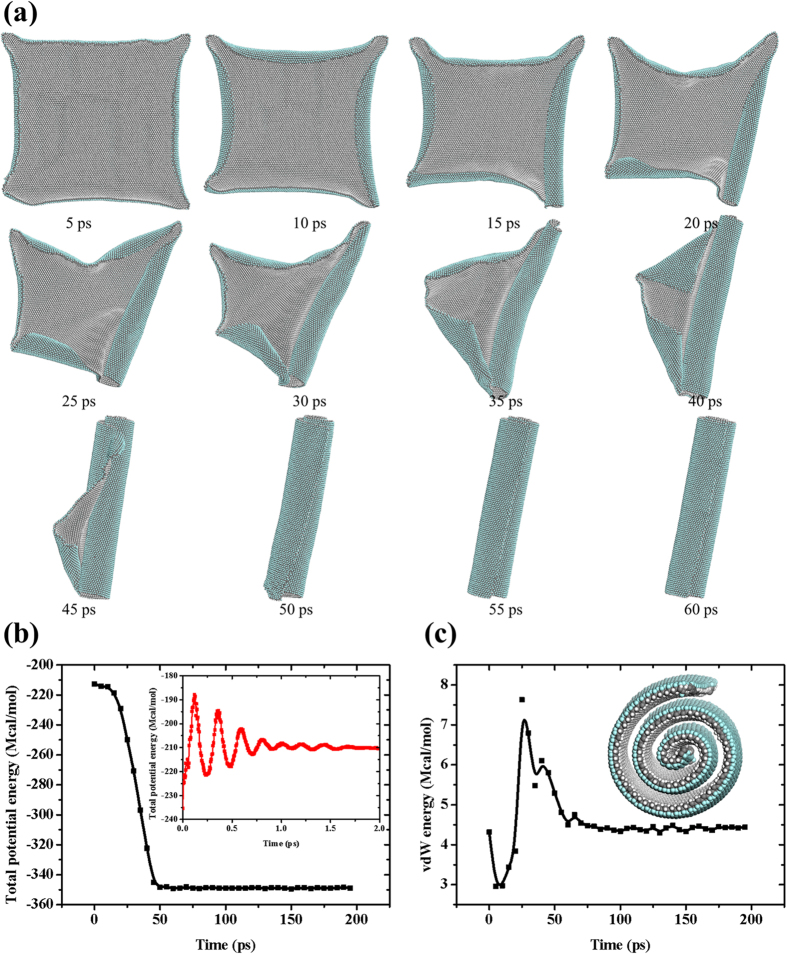
(**a**) Snapshots of the self-assembly of J-GN. (**b**,**c**) The evolutions of total potential energy (**b**) and vdW energy (**c**) versus time. The inserts are the enlarged images of the total energy from t = 0–2 ps and the side view of stable structure in the final, respectively.

Our simulation results reveal the self-assembly of hydrofluorinated J-GN at room temperature. In Fig. 2(a), it provides the snapshots of the self-assembly of J-GN with size of 211.349 Å (W) × 210.754 Å (L) (see Video S1 in the Supplementary data for the detailed self-assembly process). Because of the long-wavelength fluctuations of 2D crystals^28^, the four edges of J-GN scrolls slightly towards the side of hydrogenation after the energy minimization, i.e., the opposite side of fluoridation. The four edges of J-GN scroll continuously towards the side of hydrogenation when the dynamic simulation starts (t = 5 ps). After 10 ps, the right edge of J-GN gradually exhibits the larger deformation, which results in the formation of a cylinder roll (t = 25 ps). Although some triangular conformation is generated at the left side, the conformation is eventually disappeared with the dynamic revolution and the individual cylinder J-NS is produced (t = 50 ps). After some little adjusting, a perfect J-NS is formed (t = 60 ps). Our simulation results indicate that the self-assembly process is very quick (60 ps). Moreover, we have examined the thermostability of J-NS (in the supporting information), which means that the structural transition of J-GN from nanoplane to nanoscroll is irreversible no matter whether the formed J-NS is cooled or heated.

In order to explore the driving force of self-assembly of J-GN, the total potential energy and vdW energy of the system are tracked in the process, as shown in the Fig. 2(b,c). The insert of Fig. 2(b) is enlarged images of the total potential energy from t = 0–2 ps, which shows the fluctuation of the system at the beginning. It is clear that the total potential energy of the system is decreasing sharply from 10 ps to 50 ps, which is the main stage of the formation of J-NS. The decrease of total potential energy of the system indicates that the self-assembly of J-GN is a process of energy decreasing in which the system gradually reaches to a more stable state. As noted, after t = 50 ps, the total potential energy reaches a minimum and remains almost constant, which indicates that the system has reached the most stable state from the view of thermodynamics. Interestingly, the evolutions of the vdW energy and the total potential energy are not synchronous in our simulation. Before t = 25 ps, long-wavelength fluctuations destroy the long-range order of 2D crystals according to the Mermin-Wagner theorem^28^, so the original 2D J-GN is crumpled randomly and its structure varies obviously. It is the changing wrinkles that lead to obvious fluctuation of the vdW energy of system. Once the curling of J-NS begins (t = 25 ps), the vdW energy of the system decreases continuously due to the continual curl and ceaseless formation of scroll. After 50 ps, the vdW energy of system remains constant on account of the completion of J-NS. It is very fascinating that there is a same level of the vdW energy between the original system (J-GN) and the stable system (J-NS). So we suggest that the vdW interaction offers the main driving force to drive the structural transition, which derived from the asymmetric doping of H/F and the corresponding asymmetric orientation of *sp*^3^ bonding. Moreover, it should not be ignored that there exists the coupling of C-H/π/C-F interaction and π/π interaction between J-GN layers after 25 ps^29^, resulted from an intermolecular interaction in the adjacent C-H…F-C and paralleled six-membered rings. The coupling interaction sustains the structural transition of the J-GN to form a multi-walled J-NS in the end. Therefore, the vdW interaction and the coupling of C-H/π/C-F interaction and π/π interaction are shown to offer the continuous driving force of self-assembly, offering a novel approach in Janus-inspired design of high-quality J-NS, which will have potential applications in novel gas sensors/storage, self-propellant motors, solid surfactants, etc.

**Figure 3:**
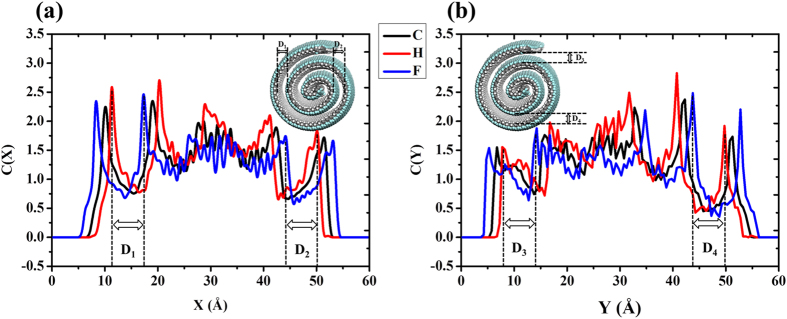
Structural analysis of the formed J-NS. The concentration profile of C/H/F atoms in X direction (**a**) and Y direction (**b**).

To study the structure of the formed J-NS, we examine the concentration profile of C/H/F atoms in X/Y directions, as shown in the Fig. 3(a,b). We define the distance between F and H of adjacent layers as the effective interlayer spacing of J-NS, instead of the distance between C and C of adjacent layers. As marked in the figure, the effective interlayer spacing of J-NS D_1_ is 6.0 Å, D_2_ is 6.0 Å, D_3_ is 6.0 Å and D_4_ is 6.0 Å, proving the good stability of the formed J-NS. On the one hand, the results are all less than 10.0 Å of the effective range of vdW interaction, which indicates that the adhesion between stacked shells is very strong and the J-NS structure is also stable. It is more favorable for the self-assembly into the J-NS structure rather than a planar J-GN. On the other hand, the distances are also larger than 3.4 Å of multi-walled carbon nanotubes (MWNTs), providing a considerable space for H_2_ storage, which will be discussed in details in the following section.

## Discussion

### The effect of geometry of J-GN on the self-assembly

**Figure 4:**
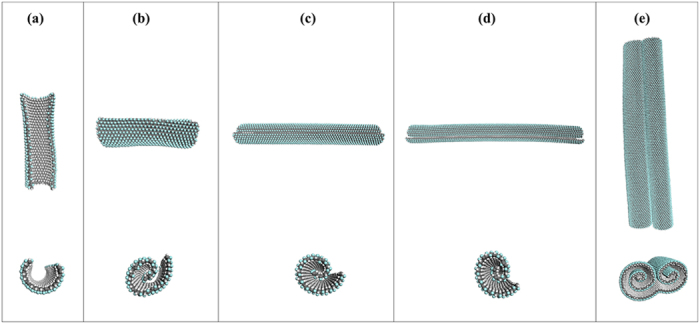
The self-assembly of J-GNs with same length of 70.16 Å and width of (**a**) 37.12 Å (**b**) 69.92 Å (**c**) 154.11 Å (**d**) 238.69 Å. (**e**) Arm-chair-like J-NS is formed from J-GN with length of 238.694 Å and width of 369.036 Å. White, gray, and green balls represent hydrogen, carbon and fluorine atoms, respectively. The upper panel is top view, and the low panel is side view.

To study the effect of geometry on self-assembly of J-GN, the self-assembly of J-GNs with various aspect ratios are further investigated. In Fig. 4(a~d), we first studied the self-assembly of 4 J-GNs with same length of 70.16 Å (armchair/L direction) but different widths of 37.12, 69.92, 154.11, 238.69 Å (zigzag/W direction), respectively. The results show that there are mainly two distinct conformational types of formed J-NS at room temperature (298 K), including arcs and J-NS structures. For a large aspect ratio (r = 1.89), J-GN only self-assembles into an arc due to the lack of sufficient driving force, as shown in Fig. 4a. Since the length edge is larger than the wide edge, there is weaker stability in the wide direction, resulting in the curly direction in the whole self-assembly. However, the driving force is not sufficient to form a nanoscroll structure, facilitating the formation of an arc with curvature radius of about 9.3 Å. As the aspect ratio of J-GN decreases, a perfect multilayered J-NS is generated in Fig. 4b (r = 1.00), which is similar with the aforementioned case in Fig. 2. When the aspect ratio of J-GN decreases continuously (Fig. 4c,d), the quadratic J-GN becomes gradually to a J-GN nanoribbon, which has a narrow shape with a smaller aspect ratio(r = 0.46, 0.29, respectively). As noted, there is some difference between the self-assembly of the aforementioned quadratic J-GN and J-GN nanoribbons. The dynamic self-assembly processes can be seen in Video S2 in the Supplementary data. Herein, the J-GN nanoribbon has negligible deformation along its width direction but remarkable curve and scrolling along the length direction, which accelerates the process of self-assembly. Therefore, the width edge dominates the self-scrolling process instead of the length edge. Interestingly, when the size continuously increases, J-GN monolayer self-assembles into an arm-chair-like J-NS, which consists of two connected J-NSs, as shown in Fig. 4e. It is the long distance between two opposite edges of J-GN monolayer that weakens the influence of initial scrolling in the two edges, which offers an opportunity of the formation of arm-chair-like J-NS.

### Self-assembly of Hydrofluorinated J-GN and Fe NWs

**Figure 5:**
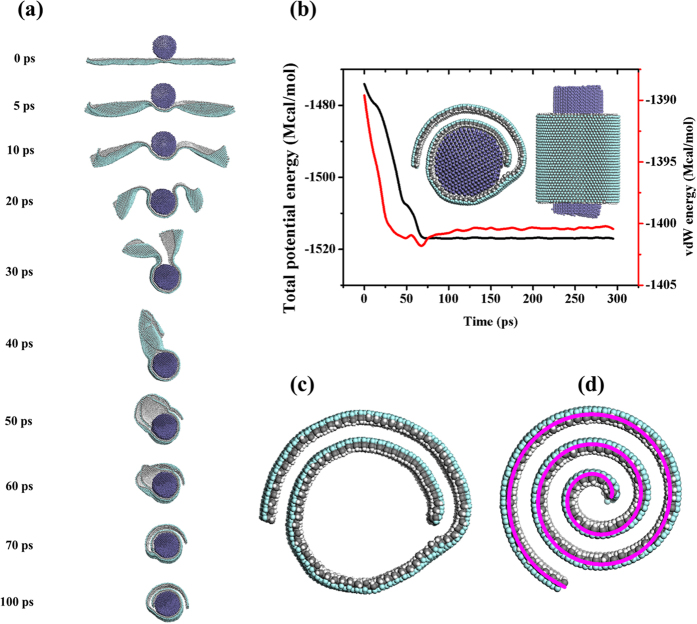
Self-assembly of Hydrofluorinated J-GN and Fe NWs. (**a**) Snapshots of the dynamic self-assembly of hydrofluorinated J-GN with size of 238.69 Å (zigzag direction) × 70.16 Å (arm-chair direction) onto a Fe NW with radius of 20.0 Å. (**b**) The evolutions of total potential energy and vdW energy versus time. The inserts are the enlarged images of final structure in top and side view, respectively. (**c**) J-NS by removing Fe NW of the self-assembly system in (**a**). (**d**) The stable structure of (**c**) after 200 ps dynamic simulation. Magenta line embedded is the fitting Archimedean spirals. White, gray, green and slate blue balls represent hydrogen, carbon, fluorine and iron atoms, respectively.

Inspired from the concept that 1-D nanomaterials are good candidate of activating the structural phase transition of 2-D nanomaterials^30^,^31^, we also investigate the interaction between hydrofluorinated J-GN and iron (Fe) nanowires (NWs). Herein, Fe NWs with magnetic performance are selected as typical representation of 1-D nanomaterials because it is facile to achieve from experimental preparation^32^. Figure 5a provides the snapshots of the dynamic self-assembly of hydrofluorinated J-GN with size of 238.69 Å (zigzag direction) × 70.16 Å (arm-chair direction) onto a Fe NW with radius of 20.0 Å (see Video S3 in the Supplementary data for the detailed self-scrolling process), which has the same size as the aforementioned J-GN monolayer in Fig. 4d. Initially, the J-GN is placed vertically to the axis of Fe NW and in the middle of Fe NW. It is clear that the attractive force between J-GN and Fe NW makes the J-GN approach to the Fe NW rapidly. Moreover, the Fe NW also prevents and dissipates the formation of J-NS along the width direction like Fig. 4d. As time goes on, the J-GN displays discontinuous wrinkles, indicating the thermodynamic instability of individual J-GN monolayer. After the J-GN attaching onto the Fe NW, the J-GN begins to curl and quickly wrap around the Fe NW to form a roll with a tail (t = 40 ps). Afterwards, the tail part begins to adjust and the right section first wraps on the Fe NW (t = 50 ps). It is found that the J-GN continues to wrap on the Fe NW and slides with a low speed along the axis of the Fe NW. Eventually, the perfect J-NS is formed at t = 70 ps and the J-NS structure holds stable all the time (t = 100 ps). Hereto, the structural transition from 2D plane to 1D tubular nanoscroll is completed with the help of Fe NW. In addition, it is inevitable that the Fe NW is also deformed because of the strong interaction between J-GN and Fe NW during the self-assembly.

In order to study the effect of the vdW interaction between J-GN and Fe NW on the structural transition of J-GN, we record the total potential energy and vdW energy of the whole system during the self-assembly, as shown in Fig. 5b. The inserts are enlarged images of final structure in top and side view, respectively. It is clearly found that the change of the total potential energy and vdW energy are almost synchronous during the self-assembly. Before 70 ps, the total potential energy and vdW energy of the system all decease sharply, which demonstrates that the self-assembly of J-GN and Fe NW is also a process of energy decreasing in which the system gradually reaches to a more stable state instead of the 2D plane. Thereafter, the total potential energy of the system reaches a minimum and remains nearly constant. Due to the adjustment of the formed J-NS, the vdW energy holds constant after slight increasing. It is worthy to noted that the vdW energy contributes to a large proportion of the total potential energy during the process of self-assembly, which suggests that the vdW force between the J-GN and Fe NW provides the main driving force to impel the self-assembly of J-GN and Fe NW. Because the vdW interaction between J-GN and Fe NW is much stronger than the vdW interaction between adjacent interlayers of J-GNs^33^, the former vdW interaction dominates the whole process of self-assembly of J-GN. This explains why the self-assembly of J-GN and Fe NW is different from the self-assembly of J-GN monolayer in the forward section.

To study the properties of the individual J-NS, we implement 200 ps dynamic simulation of the system where Fe NW is removed after the self-assembly of J-GN onto Fe NW. Intriguingly, the individual J-NS does not unfold and holds the nanoscroll structure constant after 200 ps dynamic simulation. It is found that the nanoscroll tightens at the center of the J-NS, as shown in Fig. 5c,d. The total potential energy of the system is −135.8482 MCal/mol, which is a little higher than the formed J-NS in the Fig. 4d (−103.5053 MCal/mol), resulting from that the J-NSs are fabricated along different directions. That is, the formed J-NS here is formed along the W direction, while the J-NS in the Fig. 4d is prepared along L direction. Because the size of J-GN is 238.69 Å (W) × 70.16 Å (L), the formed J-NS here has much more layers than the J-NS in the Fig. 4d, leading to a higher total potential energy of the system. However, the vdW energy of system is 1.0358 MCal/mol, which is smaller than the formed J-NS in the Fig. 4d (1.6039 MCal/mol). The results demonstrate that the J-NS formed in the presence of Fe NW is more stable than that formed in the absence of Fe NW. Furthermore, we also characterize the structure of J-NS from a topological point of view. It has been demonstrated that carbon scrolls derived from GN can be considered as sheets rolled up into Archimedean spirals^26^,^34^. From a mathematical point-of-view, the polar equation of Archimedean spirals is 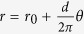, where *r*_0_ is a given core radius, *d* is the interlayer spacing, and *N* is the number of turns (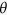 varies from 0–2*πN*). We can conclude that the formed J-NS also meets Archimedean spirals from the comparison between the J-NS and the fitting Archimedean spirals, as shown in Fig. 5d. Herein, the results declare that core radius *r*_0_ is about 4.8 Å, and the interlayer spacing *d* is 9.6 Å. It is noted that the interlayer spacing is larger than the aforementioned effective interlayer spacing D_1_~D_4_ because of the existence of H and F atoms. The perfect topological structure of J-NS has a good agreement with many nature phenomena, which also indicates the formed J-NS is stable.

### Possible applications in the field of hydrogen storage

**Figure 6:**
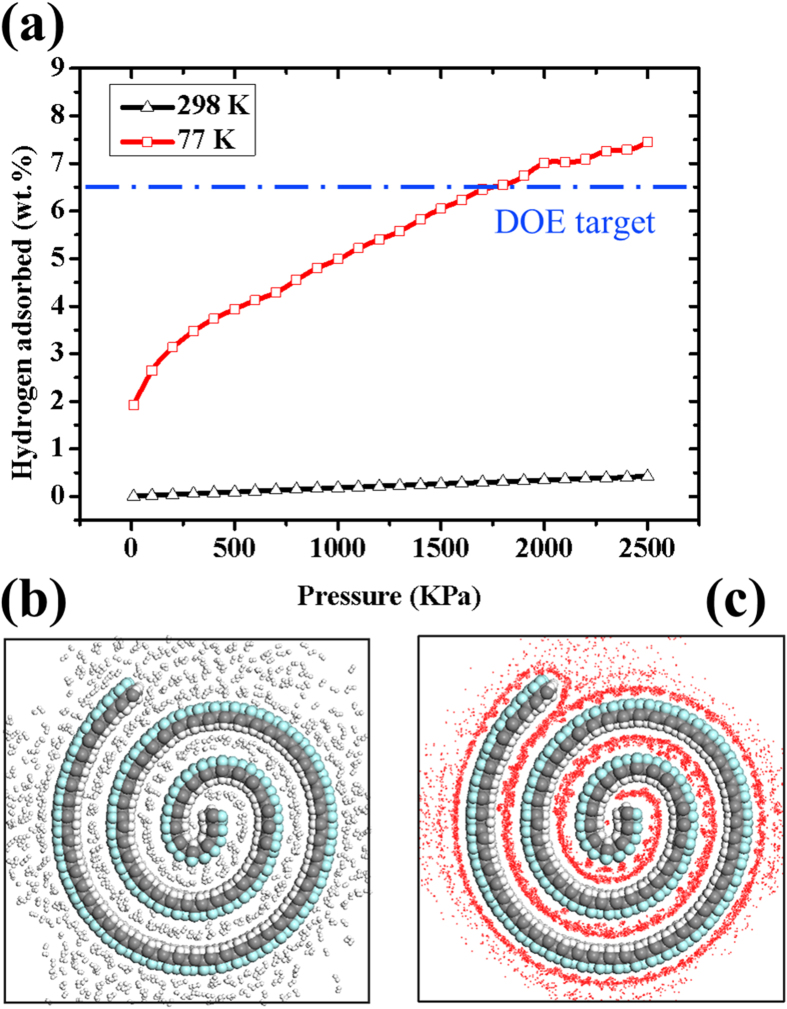
Hydrogen sorption of the formed J-NS in Fig. 2. (**a**) Sorption isotherms of H_2_ in formed J-NS at 77 K and 298 K. (**b**) Top-view snapshots and (**c**) density field of the equilibrium state of hydrogen sorption in the isolated J-NS at 77 K and 1800 KPa. White, gray and green balls represent hydrogen, carbon and fluorine atoms, respectively.

Hydrogen is recognized as the cleanest, sustainable and renewable energy carrier, which is considered as a promising candidate to replace current fossil-fuel-based energy systems^35^,^36^. Carbon-based nanomaterials have been intensively investigated from both theoretical and experimental aspects, including carbon nanotubes, graphite, activated carbon and CNS^37^,^38^. It has been demonstrated that nanoscroll structure enhances uptake and release of hydrogen because of the existence of open-ended tubular geometry differing from carbon nanotubes^37^. There are still open issues on how to increase the interlayer spacing of nanoscrolls. Herein, the effective interlayer spacing of the formed J-NS is about 6.0 Å, which is about 2 times of the diameter of hydrogen molecule and also larger than the interlayer spacing of multi-wall carbon nanotubes, so we predict the hydrogen uptake onto the J-NS. In Fig. 6a, we present the sorption isotherms of H_2_ in formed J-NS at 77 K and 298 K. Clearly, we achieve the target of the US Department of Energy (DOE) for hydrogen storage (6.5 wt % of a gravimetric capacity of stored H_2_/system weight). It can be concluded that the adsorbed H_2_ in J-NS at 77 K is much higher than those at 298 K at the same pressure. Moreover, hydrogen storage at 77 K is much more superior to that at 298 K as the pressure increases. These results suggest that the temperature can be used as a parameter to dominate the charging/releasing process of hydrogen stored in J-NS. The configurations and density field of hydrogen sorption in isolated J-NS at 77 K and 1800 KPa are shown in Fig. 6b,c. It is confirmed that the J-NS is filled completely with hydrogen molecules at low temperature (77 K). Therefore, the formed J-NS is considered as a promising candidate for hydrogen adsorption and storage by manipulating the temperature of system.

In summary, we demonstrate by molecular dynamic simulations that hydrofluorinated J-GN can self-assemble into various J-NSs at room temperature, including arc, multi-wall J-NS and arm-chair-like J-NS, by manipulating its original geometry (size and aspect ratio). It is proven that the vdW interaction and the coupling of C-H/π/C-F interaction and π/π interaction offer the continuous driving force of self-assembly of J-GN. Moreover, we also investigate self-assembly of hydrofluorinated J-GN and Fe nanowires (NWs), suggesting that Fe NW is a good alternative to activate J-GN to form J-NS. Differently, the strong vdW interaction between J-GN and Fe NW provides the main driving force of the self-assembly. Finally, the hydrogen sorption onto the formed J-NS with a considerable interlayer spacing of 6.0 Å is studied, which reaches the US DOE target, indicating that J-NS is a promising candidate for hydrogen storage by controlling the temperature of system.

Our theoretical results provide a versatile route for designing novel J-NS from 2D Janus nanomaterials for the first time. These discoveries are of great significance in the further exploration of the novel properties and practical applications of J-NS in many fields involving physics, chemistry, energy and environment, etc. Except the potential applications in H_2_ storage, the various controllable configurations of J-NSs may also possess a considerable band gap and electronic transport, which would pave the way for the development of nanoelectronics.

## Methods

### Molecular dynamics (MD) simulations

In this work, the molecular dynamics (MD) simulations were carried out by the Discover module, as implemented in the Materials Studio software package of Accelrys Inc. The force field of condensed-phased optimized molecular potential for atomistic simulation studies (COMPASS) is used to describe the interatomic interactions^39^, which is a universal all-atom force field for atomistic simulation of organic molecules, small molecules and polymers. COMPASS is a first ab initio force field that has been parametrized and validated using condensed-phase properties by ab initio and empirical data, which has been proven to be applicable in studying the self-assembly of GN and its derivatives monolayer^27^,^31^,^32^. In our MD simulations, an NVT ensemble was performed at room temperature of 298 K. In order to control the thermodynamic temperature and generate the correct statistical ensemble, we imposed Nose method in the thermostat, which ensured the invariance of the thermodynamic temperature by allowing the simulated system to exchange energy with a “heat bath”. The time step in MD simulation was 1 fs, and data were collected every 1 ps to record the trajectory of all atoms. As noted, all the simulations were calculated long enough, which ensures that the systems reached an equilibrium state. Moreover, the van der Waals interactions were calculated by atom-based method within a cutoff distance of 9.5 Å, and the Ewald summation method was employed to calculate the electrostatic interactions^40^.

### Grand canonical Monte Carlo (GCMC) simulations

In our work, the grand canonical Monte Carlo (GCMC) simulations were carried out by the Sorption module, which is implemented in the Materials Studio software package of Accelrys Inc. And gas-phase fugacities for hydrogen are calculated with the Peng-Robinson equation of state. We have used the universal force field (UFF) to describe the interatomic interactions^41^, which can accurately predict light gases adsorption, diffusion and separation in porous materials^42^. Moreover, the previous results have verified that UFF is also applicable for the hydrogen sorption and diffusion in carbon nanotubes^43^. In GCMC simulations, the van der Waals interactions were calculated by atom-based method within a cutoff distance of 15.5 Å, and the electrostatic interactions were calculated by the Ewald summation method^40^. Moreover, the Metropolis Monte Carlo methods are used, including trials of creation, destruction, regrowth, rotation, translation^44^,^45^. Equilibration duration of 5 × 10^6^ time steps is carried out in the GCMC simulation. Afterward, 5 × 10^7^ time steps of GCMC simulations are performed for production. The adsorption isotherms were calculated by simulating the average number of gas molecules at different sets of bulk pressures at the constant temperature and volume^46^.

## Additional Information

**How to cite this article**: Jin, Y. *et al.* Self-Assembly of Hydrofluorinated Janus Graphene Monolayer: A Versatile Route for Designing Novel Janus Nanoscrolls. *Sci. Rep.* **6**, 26914; doi: 10.1038/srep26914 (2016).

## Supplementary Material

Supplementary Information

Supplementary video S1

Supplementary video S2

Supplementary video S3
