# Supplementary material for: Self-Assembly of Hydrofluorinated Janus Graphene Monolayer: A Versatile Route for Designing Novel Janus Nanoscrolls
Source: Sci Rep. 2016 May 31;6:26914. doi: 10.1038/srep26914 (PMC4886628; doi:10.1038/srep26914)
Supplement: Supplementary Information [file srep26914-s1.pdf]

Supporting Information of

Self-Assembly of Hydrofluorinated Janus Graphene Monolayer: A Versatile Route for Designing Novel Janus Nanoscrolls

Yakang Jin,[†,‡] Qingzhong Xue,*[,†,‡] Lei Zhu[‡], Xiaofang Li[†], Xinglong Pan[†],

Jianqiang Zhang[‡], Wei Xing[†], Tiantian Wu[‡], and Zilong Liu[#]

[†] *State Key Laboratory of Heavy Oil Processing, China University of Petroleum, Qingdao 266580, Shandong, P. R. China*

[‡] *College of Science, China University of Petroleum, Qingdao 266580, Shandong, P. R. China*

[#] *Nano-Science Center and Department of Chemistry, University of Copenhagen, Copenhagen, DK-2100, Denmark*

Corresponding author:

*E-mail: xueqingzhong@tsinghua.org.cn; (Prof. Q. Z. Xue)

The thermostability of J-NS. In order to examine the thermostability of proposed Janus nano-scroll (J-NS), after finishing the self-assembly of Janus graphene (J-GN), we performed 200 ps NVT molecular dynamics simulations at high temperature (350 K) and low temperature (250 K), respectively. As shown in Figure S1(a), when the initial temperature is 350 K, the total potential energy and vdW energy of J-NS almost keep constant. This indicates that the proposed J-NS is extremely stable at high temperature. When the formed J-NS is cooled, we can conclude from Figure S1(b) that the proposed J-NS is also stable at low temperature. Further, the structure of J-NS (the inserts in Figure S1) indeed remains unchanged no matter whether the formed J-NS is cooled or heated, which also demonstrates the thermostability of proposed J-NS.

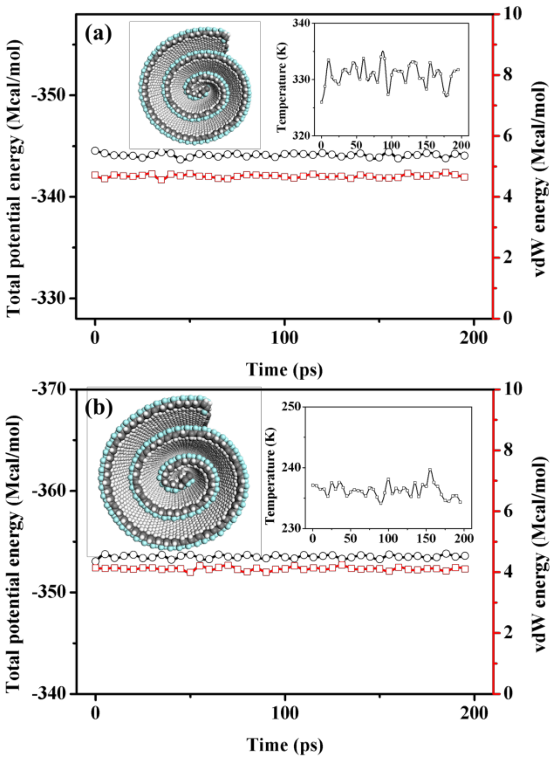

Figure S1. The evolutions of total potential energy and vdW energy versus time at high temperature (a) and low temperature (b). The inserts are the perspective view of

J-NS in the process and the plot of the temperature of system versus time, respectively.

Video legends. The Videos shows self-assembly of J-GN into J-NS structures, including Video S1: self-assembly of J-GN (a quadratic shape, 211.349 Å × 210.754 Å); Video S2: self-assembly of J-GN (a narrow shape, 154.11 Å×70.16 Å); Video S3: self-assembly of J-GN with size of 238.69 Å (zigzag direction) × 70.16 Å (arm-chair direction) onto a Fe NW with radius of 20.0 Å.
